# Irrigants and irrigation activation systems in Endodontics

**DOI:** 10.1590/0103-6440202305577

**Published:** 2023-10-27

**Authors:** Brenda P. F. A. Gomes, Emelly Aveiro, Anil Kishen

**Affiliations:** 1 Department of Restorative Dentistry, Division of Endodontics, Piracicaba Dental School - State University of Campinas, UNICAMP, Brazil; 2 Department of Dentistry, University of Toronto, Canada

**Keywords:** root canal irrigants, sodium hypochlorite, chlorhexidine, ultrasonics, photochemotherapy

## Abstract

Root canal infections are typically polymicrobial and involve strong bacterial interactions. The goal of endodontic treatment is to remove infected content from the root canal system to allow the healing of a pre-existing periapical lesion or to prevent infection of the periradicular tissues. Instrumentation alone is not capable of touching all of the root canal walls. Therefore, the irrigation process is an essential step in the endodontic treatment. However, due to the complex anatomy of the root canal system, this cleaning is very challenging. Although syringe and needle irrigation associated with the use of chemical substances is still the most used method, it does not guarantee optimal cleaning of the root canals. As a result, not only alternative irrigating substances but also numerous activation systems - which are technologies that aim to optimize the action of irrigating substances, both chemically and physically - have been developed. This work aimed to review the characteristics of both classic and current alternatives of irrigating substances and irrigation activation systems.

## Resumo

As infecções dos canais radiculares são tipicamente polimicrobianas e envolvem fortes interações bacterianas. O objetivo do tratamento endodôntico é remover o conteúdo infeccioso do sistema de canais radiculares, a fim de permitir a cicatrização de uma lesão periapical pré-existente ou prevenir a infecção dos tecidos perirradiculares. A instrumentação por si só não é capaz de tocar todas as paredes dos canais radiculares, desta forma a irrigação é uma etapa essencial no tratamento endodôntico. No entanto, devido à complexa anatomia do sistema de canais radiculares, essa limpeza é muito desafiadora. Embora a irrigação convencional com seringa e agulha, fazendo o uso de substâncias auxiliares seja o método mais utilizado, ela não garante a limpeza ideal dos canais radiculares. Como resultado, foram desenvolvidos inúmeros sistemas de ativação que visam otimizar a ação das substâncias irrigadoras, tanto química quanto fisicamente. O objetivo deste artigo foi revisar as características das substâncias irrigadoras (clássicas e alternativas) e dos sistemas de ativação destas substâncias.

## Introduction

Chemomechanical preparation aims to remove the pulp tissue, whether inflamed or necrotic/infected, creating an optimally shaped root canal space for the delivery of antimicrobial agents, disrupting bacterial biofilms, and reducing or if possible, eliminating all intracanal microbiota, while facilitating the placement of root-filling materials ^1^,^2^,^3^.

Mechanical instrumentation alone, without the use of antiseptic irrigants, already considerably reduces the bacteria present in the root canal, both by mechanical action and by exposure to oxygen, since many anaerobic bacteria have low oxygen tolerance ^4^. Byström and Sundqvist ^4^ reported that manual instrumentation using saline as an irrigant reduced bacterial cells from 10^4^-10^6^ to 10^2^-10^3^ cells (53.3% reduction). With the improvement of rotary instrumentation, the reduction of bacteria is around 98% using saline as an irrigating solution (5).

However, the root canal morphology presents distinct complexities, which include lateral and accessory canals, isthmuses, apical deltas, and dentinal tubules. These complexities render root canal cleaning an extremely challenging procedure, resulting in substantial unprepared areas with residual bacterial biofilms in the root canal ^1^,^2^,^3^.

Even in small and/or rounded canals, micro-tomographic studies report that different instrumentation systems leave approximately 10% to 50% of the total surface area unprepared. These numbers can be even higher when only the apical surface of the canal is evaluated. In more complex canals such as oval/flat canals, the amount of intact surface area after preparation can vary from 10% to 80% ^2^.

The intracanal microorganisms that persist in the intracanal and uninstrumented portion of the root canal and/or microbes that recolonize the previously filled root canal system, are considered as the main cause of persistent or secondary apical periodontitis ^1^.

In addition, a smear layer is produced on the walls of the instrumented root canal. It is composed of inorganic and organic constituents from dentinal filings and pulp tissue debris. The smear can be penetrated by bacteria, while offering protection to the biofilms that are adhering to the root canal walls and interfering with the adaptation of endodontic cements to the dentin walls. Therefore, the primary goal of endodontic treatment should be to optimize root canal disinfection and prevent reinfection ^1^. These factors emphasize the importance of root canal irrigation to remove debris, bacteria, toxic products and substrates necessary for bacterial growth from the inaccessible and uninstrumented surfaces ^6^.

To improve the antimicrobial capacities, innovative approaches in irrigants and irrigation techniques have been proposed. The aim of this work was to review the characteristics of both classic and current alternatives of irrigating substances, and the irrigation activation systems.

### Irrigants

### 1 Ideal properties of root canal irrigants

The liquid chemicals used to disinfect root canals are called irrigants. The ideal properties essential for a root canal irrigant are listed in Box 1. Several chemicals have been suggested as root canal irrigants. However, no single irrigant has all these desirable properties ^1^, ^3^, ^7^, ^8^, ^9^, ^10^, ^11^, ^12^, ^13^.

**Box 1:**
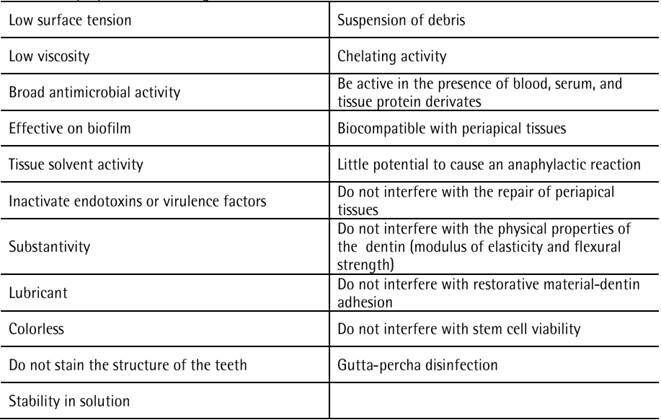
Ideal properties for an irrigant

### 2. Types of Irrigants

The process of delivering irrigation into the root canal is called irrigation. Root canal irrigation plays an important role in endodontic treatment and has two objectives. (A) Physical objective: Aims to promote the flow of irrigant through the entire root canal system while inducing sufficient physical interaction with the root canal walls for efficient debridement. (B) Chemical objective: Aims to disrupt bacterial biofilms, inactivate endotoxins, and dissolve tissue debris as well as smear layer on the canal walls ^14^. Based on these two objectives, root canal irrigants can be categorized as inactive (inert) and active irrigants.

### 2.1. Inert Irrigants

They are liquids for rinsing purposes only. Nonetheless, it is important to recognize that regardless of the chemical characteristics of the irrigant, intracanal microbial loads are reduced by the mere mechanical action of irrigation (flow and backflow) ^5^, ^14^, ^15^, ^16^, ^17^, ^18^.

### 2.1.1 Distilled water

Water is not a suitable endodontic disinfectant; however, it has an effective rinsing effect. Water can lyse bacteria that lack cell walls through a hypotonic action. However, bacteria found in the root canals often have cell walls ^15^. When distilled water is associated with most chemicals used within root canals (Sodium hypochlorite (NaOCl) in different concentrations and 2% chlorhexidine (CHX) solution/gel), there is no formation of precipitates. Therefore, distilled water seems to be the most suitable irrigant for intermediate rinses to remove traces of the chemical irrigant employed previously ^19^. It is important to remove any traces of chemicals used within the root canal in order to avoid any interaction between them. The by-product formed through such chemical interaction can be a solid precipitate that can occlude the dentinal tubules, forming a barrier between the root-filling material and the dentin surface, attributing to coronal leakage. Furthermore, the by-products formed may also be toxic to periapical tissues ^19^.

### 2.1.2 Saline solution

Despite its great biocompatibility _^16^_ , the use of saline is not recommended as the main irrigating solution due to the lack of antimicrobial and tissue dissolution properties ^16^. However, it must be highlighted that numerous studies did the root canal preparation with an inert irrigant (saline solution) as a control group and obtained a great percentage of bacterial reduction with no differences among the antimicrobial substances tested ^5^,^17^,^18^. The mechanical action of the endodontic instruments associated with the physical action (flow and backflow) of the root canal irrigation seems also to play a role in the microbial and endotoxin removal from infected root canals ^17^,^18^,^20^,^21^.

Saline solution when used as an irrigant, does not form a precipitate with NaOCl, yet it does produce a salt precipitate when associated with 2% CHX both in gel and solution forms. The precipitate formed between CHX and saline solution is attributed to the salting-out process, wherein the application of saline solution increased the salt concentration and precipitated the CHX salts ^19^.

Regarding the use of ultrasonic activation, the differences in the physical properties of water can influence the transmission of ultrasound energy to the irrigant. The bubbles formed in salt water tend to be more numerous (particularly the smallest bubbles) and are less prone to coalesce than bubbles in fresh water. Vapor could diffuse into the bubble during bubble expansion and the bubble dynamics depend on the concentration of the gas dissolved in the liquid, the temperature of the liquid, and amounts of surface-active impurities. Therefore, PUI with sterile saline removes significantly more planktonic bacteria than syringe irrigation of saline although saline does not dissolve organic tissue and is not bactericidal. However, with water, there is no significant difference between PUI and syringe irrigation regarding the removal of dentin residues or planktonic bacteria ^22^.

### 2.2 Active Irrigants

They can be classified as chemical and natural agents. Chemical agents presented different properties such as tissue solvent action (NaOCl and ClO₂); bactericidal (CHX, NaOCl), bacteriostatic (MTAD) action; and mild (HEBP) and strong chelating action (EDTA). Natural agents (green tea, Triphala) have been considered due to their antibacterial activity ^7^.

### 2.2.1 Sodium hypochlorite (NaOCl)

Sodium hypochlorite (NaOCl) has a long history of use in medicine and dentistry. During the First World War, the chemist Henry Drysdale Dakin and the surgeon Alexis Carrel extended the use of a buffered 0.5% NaOCl solution to irrigate infected open wounds (burns) ^7^. It was assumed that in the confined space of a root canal system, higher concentrations should be used as they would be more efficient than Dakin's solution ^1^. Currently, NaOCl solutions remain the most widely used solutions to irrigate root canals during endodontic therapy, mainly due to their unique tissue solvent effect and antibiofilm action ^12^. The effectiveness of NaOCl in the root canal is strongly related to the volume and frequency of irrigation ^23^. Although there are numerous studies, there is no consensus about the concentration to be used for root canal treatment ^23^.

Sodium hypochlorite is a strong oxidizing agent. The level of available free chlorine determines the reactivity of sodium hypochlorite. Nonetheless, the available chlorine concentration in sodium hypochlorite deteriorates with time, exposure to light/heat, and on contact with air, metals, metallic ions as well as organic materials ^24^. Chlorine affects a broad range of microbes including viruses and fungi, while oxygen kills anaerobic bacteria. The dissolution of necrotic pulp tissue and organic debris is achieved by the proteolytic effect of free chlorine ^14^. Reactive chlorine in an aqueous solution at body temperature can essentially take two forms: hypochlorite (OCl−) or hypochlorous acid (HOCl). The state of available chlorine depends on the pH of the solution. Above a pH of 7.6 the predominant form is hypochlorite, below this value it is hypochlorous acid. Both forms are extremely reactive oxidizing agents. The pure hypochlorite solutions, as used in endodontics, have a pH of 12, and therefore all available chlorine is in the form of OCl−. However, at identical levels of available chlorine, hypochlorous acid is more bactericidal than hypochlorite. One way to increase the effectiveness of hypochlorite solutions would be to lower the pH. Finally, the caustic potential of hypochlorite solutions appears to be influenced primarily by the available chlorine rather than pH or osmolarity ^1^.

Advantages:

- Economical solutions that are easily obtained with a good shelf life ^14^, ^25^.
- Lubricating agent ^9^,^26^
- Bleaching effect on blood and blood-stained dentin ^12^.
- Broad-spectrum antibacterial activity. The duration of interaction (irrigation) with NaOCl, influenced its antibacterial efficacy ^23^. Higher concentrations and warm solutions will also increase the ability of hypochlorite to penetrate dentinal tubules ^6^.
- Tissue solvent action ^12^. Some factors can interfere with the organic tissue dissolution capacity of NaOCl. Longer exposure time, higher concentrations, higher solution temperature, and the use of an activation system facilitatefacilitate the tissue dissolution properties of hypochlorite ^1^,^6^,^27^. Additionally, ultrasonic agitation of NaOCl produces heat, accelerating its chemical reactivity, which in turn increases its ability to dissolve collagen ^1^, ^6^, and produces a greater reduction in microbial load within root canals ^28^.
- It is the only irrigant with the ability to disrupt biofilms ^9^,^12^. Biofilm is a community of surface-bound microorganisms embedded in an extracellular matrix of polysaccharides. This growth mode allows bacteria to survive in a hostile environment ^9^. Hypochlorite can cause different effects on resident bacteria in a biofilm: (a) Complete dissolution of cells with no visual evidence (b) Bacterial cells are dislodged from the biofilm matrix and are nonviable (c) Bacterial cells remain adherent within the biofilm but are nonviable (d) Bacterial cells are dislodged from the biofilm, but are viable (e) Bacterial cells remain adherent to the biofilm and are still viable ^7^.
- Ability to inactivate endotoxins [Lipopolysaccharide (LPS)]. LPS is a component of the outer membrane of Gram-negative bacteria that is an important mediator in the pathogenesis of apical periodontitis. It is known to intensify the sensation of pain in endodontic infections. The effectiveness of 2.5% NaOCl to inactivate endotoxin is lower when compared to intracanal medication based on calcium hydroxide ^1^. However, 5.25% NaOCl or calcium hydroxide from 24 h to 30 days are equally effective in neutralizing endotoxins ^21^.
- Disinfecting agent for gutta-percha points. NaOCl can kill vegetative forms within a short period. However, it is not able to eliminate some spores. As a strong oxidizing agent, 5.25% NaOCl can cause local changes in the surface roughness of gutta-percha cones. In addition, crystals may form on the surface of the gutta-percha points after rapid sterilization with 2.5% and 5.25% NaOCl, demonstrating that the final rinse with distilled water is essential. NaOCl leads to an increase in the free energy surface (wettability) of the gutta-percha points, interfering positively with the adhesion mechanism ^9^.

Disadvantages: Despite these excellent properties, hypochlorite has several inherent disadvantages, which are listed in the following:

- Unpleasant smell and taste ^14^
- Extreme corrosiveness to metals ^14^
- Unstable in solution. Chlorine, which is responsible for the dissolution and antibacterial capacity of NaOCl, is unstable and is rapidly consumed during the first phase of tissue dissolution. Thus, continuous replenishment of NaOCl is essential ^22^.
- High toxicity. It is a potential irritant of periapical tissues, especially in high concentrations if extruded beyond the apex ^23^. In contact with vital tissues, NaOCl rapidly oxidizes the surrounding tissues, leading to rapid hemolysis and ulceration, which are directly caused by the chemical burn-mediated inhibition of neutrophil migration and damage to the endothelial and fibroblast cells ^29^,^30^. Sudden pain, profuse bleeding, inflammation, ecchymosis, hematoma, almost immediate swelling, and sometimes even necrosis and paresthesia constitute a triad of signs/symptoms pathognomonic of NaOCl extrusion ^30^,^31^. All or most of the signs and symptoms are resolved within a few weeks. Permanent sequelae could be divided into nerve lesions and scar tissues. Neurologic examination of the trigeminal and facial nerves should be performed once the anesthesia has dissipated ^30^. The intensities of the side effects depend on the concentration and volume of extruded NaOCl ^29^.
- Inability to remove smear layer ^14^. NaOCl lacks the ability to remove the inorganic part of the smear layer. Hence, a combination of NaOCl and EDTA (chelating agent) is recommended to remove the smear layer ^1^.
- Reduced bonding to dentin ^14^. The reduced bond strength seen between adhesive systems and dentin walls may occur due to the removal of collagen fibrils from the dentin surface by NaOCl, which may prevent the formation of a consistent hybrid layer ^9^. Another reason is that the remnants and oxidation by-products of NaOCl exhibit a negative effect on the polymerization of dental adhesive systems. The compromised bond strength with NaOCl-treated dentin could be restored by the application of an antioxidant solution before the adhesive procedure, resulting in neutralization and reversal of the oxidizing effect of NaOCl treatment on the dentin surface ^32^. Depending on the concentration and application time, the use of sodium thiosulfate (Na₂S₂O₃) can restore the bond strength to NaOCl-treated dentin. The use of sodium thiosulfate can significantly increase the bond strength of composite resin to dentin treated with NaOCl, allowing adhesive restorations to be applied immediately after endodontic treatment ^32^.
- Reduction in the mechanical properties of dentin, such as its modulus of elasticity, resistance to flexion, and microhardness, as NaOCl is a non-specific oxidizing and proteolytic agent, it oxidizes the organic matrix and denatures the collagen components of the smear layer. This effect is time and concentration-dependent. This removal of dentin organic components by NaOCl, altering its properties, can result in fracture of endodontically treated teeth ^1^, ^8^, ^13^.
- Reduced bond strength between adhesive systems and dentin walls. This reduction may occur due to the removal of collagen fibrils from the dentin surface by NaOCl, preventing the formation of a consistent hybrid layer ^7^.
- Challenges in regenerative endodontic procedures ^14^. It interferes with stem cell attachment to dentin and abrogates the ability of dentin-based growth factors to effectively mediate dentin-pulp regeneration ^33^. The use of the maximum concentration practiced clinically leads to greatly reduced stem cell survival and loss of odontoblast-like cell differentiation ^26^. Fortunately, these effects of NaOCl could be moderated and/or ameliorated by the application of 17% EDTA as a final irrigation, particularly if the original concentration of NaOCl is 1.5% ^33^. Due to the good biocompatibility of saline solution, it is suggested as a final rinse after irrigation with NaOCl to help promote the adherence of DPSC (dental pulp stem cell) on root canal dentin ^16^.

### 2.2.2 Chlorhexidine (CHX)

CHX is a nearly colorless to pale straw-colored or slightly opalescent, odorless or almost odorless substance. It is widely used in dentistry and is considered the gold standard for antiseptic. The most used concentrations as mouthwashes are 0.12 and 0.2%. In Endodontics, it has been proposed as a promising irrigation agent to replace NaOCl during root canal disinfection and endodontic instrumentation at a concentration of 2% ^9^,^23^.

It consists of a strong base and is more stable in salt form. The original salts were chlorhexidine acetate and hydrochloride, both of which were relatively poorly soluble in water. In 1957, they were replaced by chlorhexidine digluconate, which is a highly water-soluble salt. Solutions prepared from all salts have an extremely bitter taste that must be masked in formulations intended for oral use. As with sodium hypochlorite solution, heating a chlorhexidine solution at a lower concentration can increase its local effectiveness in root canals, while maintaining low systemic toxicity ^1^,^9^,^25^.

It can be purchased commercially or in compounding pharmacies, either in liquid or gel form. The gel form consists of a gel based on 1% natrosol + chlorhexidine gluconate, in an optimal pH range of 5.5 to 7.0. Natrosol (hydroxyethylcellulose, pH 6-9) is a water-soluble, carbon polymer. Hence, it is easily removed from the root canal with a final rinse with distilled water. The gel form has advantages over the liquid form, as it lubricates the root canal walls while reducing the friction between the endodontic file and the dentin surface. This facilitates instrumentation, decreasing the risk of instrument fracture besides improving the removal of organic tissue, which to a certain extent compensates for its inability to dissolve them. Furthermore, chlorhexidine diminishes the formation of a smear layer, keeping almost all dentinal tubules open. The viscosity of chlorhexidine gel is suggested to keep the debris in suspension (rheological action), a fact that does not occur with the liquid formulation. Another advantage is that the gel formulation can keep the "active principle" of CHX in contact with microorganisms for a longer time, inhibiting their growth ^9^.

Chlorhexidine can be applied in all phases of endodontic treatment, including surface disinfection of the operative field, during root canal orifice enlargement, during the removal of necrotic tissues, before performing the root canal length determination, during instrumentation (chemo-mechanical preparation), before patency filing, as intracanal medication alone or combined with other substances and shaping of gutta percha points, in the removal of gutta percha during retreatment, in the disinfection of the prosthetic / post space, etc. ^9^.

A 24G or smaller needle is indicated for its deposition in the root canal. Its protocol for use during instrumentation consists of the deposition of 1mL of CHX gel before placing the file, followed by a rinse with 5mL of distilled water to irrigate the canal ^9^. It is noteworthy that before using EDTA or any other chemical, it is necessary to remove any traces of CHX through a final flush with 10 mL of distilled water ^9^,^19^.

Advantages:

- Lack of foul smell and bad taste ^11^
- Retains its activity in the presence of blood, wounds, and burns ^34^ and organic matter ^35^
- Highly effective antimicrobial against Gram-positive and Gram-negative bacteria, facultative and strict anaerobes, yeasts, fungi (mainly *Candida albicans*), and some types of viruses (respiratory viruses, herpes, cytomegaloviruses, HIV). However, it is inactive against bacterial spores at room temperature ^9^,^23^,^25^. The antimicrobial activity is pH dependent, with the ideal range being 5.5-0.7, which falls within the pH of body surfaces and tissues ^7^, ^9^. Furthermore, it retains its antimicrobial activity even in the presence of blood and other organic matter ^9^, ^25^. The effectiveness of CHX is due to the electrostatic interaction between its positive charge and the negatively charged molecules (phosphate groups) on the microbial cell walls, altering the osmotic balance and resulting in cell lysis ^7^.
- Effective against bacterial biofilms. Bacteria present in the biofilm differ greatly in phenotype when compared to their planktonic forms, and are much less susceptible to antimicrobial death. Although CHX is effective against bacterial biofilms it did not disrupt biofilm structures ^9^.
- Substantivity is the property that results from adsorption or deposition of CHX on negatively charged surfaces in the mouth, such as enamel, dentin, cementum, mucosa, and restorative materials, and is slowly released from these retention sites, thus maintaining prolonged antimicrobial activity ^3^,^23^. The antimicrobial substantivity depends on the number of CHX molecules available to interact with tissue surfaces ^7^,^9^.
- Lower cytotoxicity. The biocompatibility of CHX at clinically used concentrations is acceptable ^7^,^11^. Therefore, it is recommended as an alternative to NaOCl, especially in cases of open apex, resorption, foraminal enlargement, root perforation, or in cases of allergy ^9^,^11^. Despite being less cytotoxic, it should not be extruded into periapical tissues, as any irrigant, regardless of toxicity, has the potential to cause problems if it is extruded into periradicular tissues ^11^.
- CHX can be used as an intracanal medication either alone or in combination with other substances. CHX alone does not act as a physical barrier and does not exhibit radiopacity. The use of CHX gel as an intracanal medication is recommended for a short period ^3^-^5^ days), in cases where the root canals were completely instrumented, but could not be filled due to lack of time or presence of exudate. On the other hand, the combination of CHX with calcium hydroxide aims to increase the antimicrobial properties of calcium hydroxide, while maintaining its biological characteristics, and mechanical properties, and acting as a physical barrier. Calcium hydroxide pastes with CHX gel, alone or with ZnO, have greater antimicrobial activity than those prepared with distilled water or saline ^9^, ^25^.
- Delay coronal leakage. Either canals medicated with CHX alone or in combination with calcium hydroxide delayed microbial ingress, due to their antimicrobial activity and substantivity. This finding is interesting for application in cases wherein the coronal restoration is defective or lost ^9^, ^36^.
- Canals irrigated or medicated with CHX do not negatively affect the ability of root fillings to prevent fluid penetration into the root canal system through the apical foramen ^9^.
- CHX is a vehicle for sodium perborate in intracoronal bleaching procedures. CHX increases the antimicrobial effect, while it does not adversely affect dentin microhardness ^9^, ^37^.
- CHX is a non-specific inhibitor of Matrix Metalloproteinases (MMPs). MMPs are enzymes that play a role in the breakdown of the collagen network in bonded restorations. During bonding procedures, resin monomers infiltrate the demineralized dentin, thus forming a structure called a hybrid layer. Unlike NaOCl, CHX does not interfere with the exposed collagen in the organic matrix of root dentin. So it aids in maintaining the quality of the dentin substrate for restorations with resin-based materials ^9^.
- Improved dentin adhesion. Inhibition of MMP may be beneficial in preserving hybrid layers through the application of a synthetic protease inhibitor such as CHX. In general, because of its broad-spectrum MMP inhibitory effect, CHX can significantly improve resin-dentin bond stability ^7^.
- Irrigation with CHX increases the bond strength to root dentin. Applying 2% CHX to cavities after acid etching and before hybridization with adhesive monomers prevents loss of bond strength over time by preserving the integrity of the hybrid layer ^9^.
- The use of CHX increases the wettability of endodontic sealers on dentin, which can be explained by the presence of surface surfactant in CHX, increasing the surface energy and promoting higher wetting ability to dentin ^36^.
- Disinfecting agent for gutta-percha points. CHX has the ability to kill vegetative forms within short periods, however it is not able to eliminate some spores. 2% CHX does not change the properties of the gutta-percha cone even after 30 minutes of exposure. Like NaOCl, the application of CHX leads to an increase in the surface free energy (wettability) of gutta-percha points that interferes positively with the adhesion mechanism. However, compared to NaOCl, the CHX application offers higher values of surface free energy. Gutta-percha points disinfected with CHX presented smaller contact angles than NaOCl, favoring better interaction between gutta-percha and sealer. CHX gel can also be used to mold gutta-percha points, which improves their adaptation to the apical dentin wall (unpublished data) ^9^.

*Disadvantages:*

- The main limitation of CHX as an endodontic irrigant is its inability to dissolve pulp tissue ^23^. Bleeding in the case of vital pulp will only stop with the thorough removal of the pulp tissue by instrumenting the root canal in its entirety, as CHX does not promote superficial necrosis. However, due to its viscosity and rheological properties, CHX gel holds the debris in suspension, promoting better residual tissue removal and mechanical cleaning of root canals ^9^.
- Inactivity on endotoxins (LPS). CHX does not possess an endotoxin neutralizing effect. However, after Ca(OH)_2_ dressing for 7 days, both 2.5% NaOCl and 2% CHX can neutralize endotoxins ^21^.
- Reactivity with other irrigating substances. The interaction between EDTA and CHX forms a white milky precipitate through an acid-base reaction that covers the dentinal tubules, which may interfere with the seal achieved in root filling ^9^, ^19^. Another chemical interaction to be considered is CHX with NaOCl. There is an irrigation regime that aims to take advantage of both solutions, using NaOCl for instrumentation, followed by EDTA and a final irrigation with CHX. Or even the use of NaOCl for instrumentation and CHX as an intracanal medication. However, the interaction between CHX and NaOCl forms para-chloroaniline (PCA), a cytotoxic orange-brown precipitate. This chemical smear layer can cause discoloration of the tooth, blockage of dentinal tubules, and affected the seal of root-filling ^7^,^9^,^19^.
- Only one adverse effect has been published concerning CHX solution as an endodontic irrigant (Khanifan et al. ^11^. However, despite being less caustic than NaOCl, 2% CHX solution can be irritating to the skin ^25^. Chronic dermatitis is a common adverse reaction. The incidence of skin irritation and hypersensitivity is low, while its biocompatibility is acceptable. CHX adverse effects are usually related to its topical or oral application, including reversible discoloration of the tongue, teeth, and restorations (silicate or composite), dysgeusia as well a burning sensation of the tongue ^7^, ^9^. The US Food and Drug Administration (FDA) announced in February 2017 that, while rare, the number of reports of severe allergic reactions to CHX skin antiseptic products has increased in recent years. FDA has identified 43 cases worldwide reported from January 1, 1969, to June 4, 2015, of anaphylactic reaction with the use of topical CHX gluconate products. Twenty-four of these cases were reported after 2010. All cases were severe: 26 reported the outcome as life-threatening, 12 required hospitalization, and 2 deaths were attributed to the anaphylactic reaction ^11^.
- Application in regenerative endodontic procedures. CHX has been shown to hinder the viability of human apical papillary stem cells ^9^. Although CHX displayed toxic effects on stem cells from apical papilla (SCAP) when applied directly or indirectly, a time short-term application of CHX and neutralization with L-α-lecithin can minimize its toxic effect on SCAP ^38^. CHX has been successfully used as an irrigant or combined with calcium hydroxide as a medicament in pulp revascularization cases. However, CHX may present challenges when used in regenerative procedures due to its toxic effects on stem cells and the ability to form toxic chemical byproducts with sodium hypochlorite ^33^. Saline solution, due to their good biocompatibility, is suggested in regenerative endodontic treatments as the final rinse following irrigation with CHX to help promote DPSC (dental pulp stem cell) adherence and proliferation ^16^.

### 2.2.3 EDTA

Dentin, pulp remnants as well as the smear layer formed within the root canals post instrumentation, can affect the antibacterial efficacy of endodontic irrigants ^14^. These components act as a substrate for bacterial metabolism, prevent optimal diffusion of disinfectants, compromise the coronal/apical seal, and serve as a pathway for recontamination ^3^. Therefore, to ensure adequate bacterial killing in an infected root canal, the irrigant used must penetrate or remove the root canal debris/smear layer ^14^. Currently, no irrigant can act simultaneously on the organic and inorganic components of the smear layer ^8^. In order to completely remove tissue debris and the smear layer, the use of antibacterial irrigants with a chelating agent is recommended. This combination will result in better cleaning and allow the irrigants and medicaments to penetrate deeper into the dentinal tubules ^39^.

Ethylenediaminetetraacetic acid (EDTA) is another agent used in contemporary clinical endodontics ^39^. EDTA demineralizes the inorganic components of dentin via calcium chelation ^8^. EDTA reacts with calcium ions in dentin and forms soluble calcium chelates. During root canal treatment, EDTA decalcifies intertubular dentin to a depth of about 20-30 µm in 5 minutes. However, its action is limited to 50 μm, even after more than 24 hours of exposure time. A continuous rinse with 5 ml of 17% EDTA as a final rinse for 3 min effectively removes the smear layer from the root canal walls, but authors also claimed that 1 min is also effective ^7^. Several different systems of mechanical activation of EDTA to improve endodontic disinfection have been proposed including manual agitation with gutta-percha cones, endodontic instruments or special brushes, vibrating systems activated by low-speed handpieces or by sonic or subsonic energy, use of ultrasonic or laser energy to mechanically activate the irrigants and apical negative pressure irrigation systems ^40^. Depending on the system used, there is a reduction in the time of the chemical substance inside the canal, from 3 min (e.g. agitation with gutta-percha, 3 cycles of 1 min) to 60-90 s (e.g. ultrasonics, 3 cycles of 20-30s), however, there is a trending of renewal of the EDTA from the root canals after each agitation (3 cycles) ^28^, ^41^.

Advantages:

- Highly biocompatibility to such an extent that it is commonly used in personal care products ^1^.
- Ability to detach root canal surface adherent bacterial biofilms ^1^.
- Antimicrobial activity ^42^, which depends on the vulnerability of the bacteria tested.
- Chelating property ^8^, which is the keystone for EDTA indirect removal of LPS adhered to root canal walls ^21^, favoring endodontic disinfection ^20^.
- Removal of smear layer. This characteristic allows the opening of dentinal tubules, allowing deeper access to irrigants, medications, and sealers, in an attempt to maximize the bactericidal/bacteriostatic effect of these agents ^43^. In addition, the presence of a smear layer on the root canal walls may interfere with the adherence and proliferation of stem cells, which can compromise therapeutic outcomes in regenerative endodontic procedures ^16^.
- Inhibition of MMP activity. EDTA and CHX can help protect the hybrid layer from degradation by inhibiting MMPs. EDTA significantly inhibits the endogenous MMP activity of human dentin within 1-2 minutes (44). Nonetheless, CHX binds very firmly to demineralized dentin and sustains MMP inhibition for much longer periods than EDTA ^44^.
- EDTA reactivity is enhanced when combined with activation systems. The activation of chelating agents, independent of the protocol used, benefits smear layer removal from root canals ^45^.
- EDTA promotes the release of growth factors from the dentin matrix which may aid in regenerative endodontic procedures ^39^, ^46^. The American Association of Endodontists and the European Society of Endodontology recommend the use of a 17% EDTA solution as a final irrigation. EDTA's primary use is as an irreversible chelating agent. It binds to calcium ions and releases root dentin growth factors that can promote the recruitment of dental stem cells to the injury site, stimulate stem cell differentiation, and promote the regeneration process ^46^, ^47^.

Disadvantages:

- EDTA shows weak antimicrobial properties by itself compared with NaOCl or CHX ^1^,^39^, which does not agree with the work of Prado et al. ^42^.
- Strong interaction with NaOCl. EDTA upon interaction with NaOCl immediately reduces the available chlorine in the solution, decreasing the reactivity of NaOCl and rendering it ineffective on bacteria and necrotic tissue. Therefore, EDTA should never be mixed with sodium hypochlorite ^1^,^13^. When NaOCl at the different concentrations was associated with 17% EDTA no precipitate was found. The association of NaOCl with 17% EDTA led to the formation of bubbles. The presence of bubbles is less intense for EDTA than for phosphoric or citric acid. These bubbles are mainly chlorine gas a toxic product. The bubble formation of chlorine gas (Cl_2_) results from an increase in proton (H^+^) concentration in the presence of chloride ions (Cl^−^), which is the usual impurity of NaOCl solutions, shifting the equilibrium toward the formation of Cl_2_. In addition, Cl2 can also be produced by the oxidation of EDTA or citric acid by HOCl. With the dilution of NaOCl, fewer undesirable products were formed. NaOCl at different concentrations and 2% CHX gel and solution, when associated with distilled water, did not form any precipitate. Thus, distilled water seems to be the irrigant more indicated to be used in intermediate rinses to remove traces of the previously used chemical auxiliary substance. NaOCl associated with EDTA, citric acid, and phosphoric acid leads mainly to chlorine gas formation. Intermediate flushes with distilled water seem to be appropriate to prevent or at least reduce the formation of by-products ^19^.
- Interacts with CHX: Interaction between CHX and EDTA results in a white milky precipitate, found to be related to the acid-base reactions ^19^. More than 90% of the precipitate mass was either EDTA or CHX salt with less than 1% of the potential decomposition product, p-chloroaniline. High recovery indicates that CHX is not degraded by EDTA under normal conditions. The precipitate is likely to be a salt formed by the electrostatic interaction between cationic CHX with anionic EDTA. However, the clinical significance of this precipitate is largely unknown ^7^ .

Table 1 shows a summary of the properties of the main irrigants used (sodium hypochlorite, chlorhexidine, and EDTA) ^1^, ^3^, ^9^, ^11^, ^13^, ^14^, ^23^, ^25^, ^39^, ^46^.

### 3.New Irrigant Alternatives

As currently none of the irrigating solutions available on the market have all the ideal characteristics for an endodontic irrigant, there is a constant search for a solution that presents as many of these desirable properties as possible, to provide a better prognosis for endodontic treatment.

### 3.1. Nanoparticle

Nanomaterial denotes a natural, incidental, or manufactured material containing particles in an unbound state or as an aggregate or as an agglomerate and where 50% or more of the particles in the number, size, distribution, one or more external dimensions is in the size range of 1-100 nm ^48^. These materials have exceptional advantages in certain clinical applications of their unique physical and chemical properties, ultra-small sizes, large surface area-to-mass ratio, and enhanced reactivity ^49^,^50^.

**Table 1:** Summary of irrigating properties

	NaOCl	CHX	EDTA
Broad antimicrobial activity	X	X	X
Action on biofilm	X	X	X
Tissue solvent activity	X		
Inactivate endotoxins or virulence factors	X		
Substantivity		X	
Lubricant	X	X	
Bleach	X		
Stability in solution		X	
Suspension of debris		X	
Chelating activity			X
Be active in the presence of blood, serum, and tissue protein derivates		X	
Biocompatible with periapical tissues		X	
Minimal potential to cause anaphylactic reaction		X	
Do not interfere with the physical properties of dentin (modulus of elasticity and flexural strength)		X	
Do not interfere with dentin adhesion		X	X
Gutta-percha disinfection	X	X	
Do not Interfere with pulp regeneration			X

Nanoparticles (NPs) are employed to design highly specific therapeutic strategies that interactinteract at the subcellular and molecular levels to provide maximal therapeutic efficacy with minimal side effects ^51^. There are different classifications for NPs based on their: (a) composition (organic or organic), (b) particle shape (particles, spheres, tubes, rods, plates, fibers, etc.), or (c) origin (naturally occurring or synthetic). There are also functionalized (conjugated) NPs, which has a core made up of one material while additional molecules, drugs, chemical, or proteins are bonded on its surface or encapsulated within it. The functionalized NPs present a unique therapeutic advantage or act as a delivery vehicle for the functionalized moiety. The characteristics responsible for the unique properties of nanoparticles are also responsible for their potential toxicity to oral tissues, systemic health, and the environment. The extent of toxicity depends on a myriad of factors such as material, concentration, duration of exposure, aggregation, particle size, geometry, and surface charge ^49^.

A systematic review concluded that the most commonly used nanoparticles in endodontics are silver nanoparticles followed by polymeric ones for disinfection ^52^. Silver nanoparticles are effective for biofilm elimination when used as a root canal irrigant/medicament. It has been shown that the antibiofilm efficacy of silver nanoparticles for root canal disinfection depends on the mode of application. The gel form is more effective than the solution form. 0.02% silver nanoparticles gel as medicament significantly disrupted the structural integrity of the biofilm and resulted in the least number of post-treatment residual viable *E. faecalis* cells compared with 0.01% silver nanoparticles gel, calcium hydroxide, and syringe irrigation with 0.1% silver nanoparticles solution. They suggested that the prolonged duration of interaction between the positively charged silver nanoparticles and negatively charged bacterial biofilm when used as a medicament for 7 days, resulted in marked destruction of biofilm structure and killing of biofilm bacteria ^53^. Gutta-percha coated with AgNPs was developed as an antimicrobial and antifungal root canal core filling material. They are also incorporated as an antibacterial material in the mineral trioxide aggregate (MTA) to enhance the success of pulp capping, apexification, and sealing of perforations ^54^.

Chitosan nanoparticles (CS NP) along with zinc oxide nanoparticles (ZnO NP) were tested for root canal disinfection ^55^. Bacteria in planktonic form were totally eliminated by both chitosan nanoparticles and zinc oxide nanoparticles. However, when tested as biofilms, they required higher concentration and longer interaction time for complete elimination. These nanoparticles retained their antibacterial properties after aging for 90 days ^55^. The proposed antibacterial mechanism for cationic nanoparticles such as chitosan nanoparticles is via electrostatic interaction between the positively charged nanoparticles and negatively charged bacterial cell membranes leading to alteration in cell wall permeability and eventually cell death ^56^, ^57^. Chitosan and antimicrobial drug-silica coassembled nanoparticles were also incorporated with the root canal sellers to enhance their antibacterial properties.

Bioactive glass received considerable interest in root canal disinfection due to its antibacterial properties. BAG consists of SiO_2_, Na_2_O, CaO_2,_ and P_2_O_5_ at various concentrations. Nanometric bioactive glass increased pH, which is mainly responsible for its antimicrobial activity. These particles also released Ca ^2+^, Na^+^, PO_4_^3-,^ and Si^4+^ which contributed to the formation of bonds with mineralized hard tissues. BAG has been used for *in vitro* root canal disinfection studies (^58^ ^,^ ^59^ ^,^ ^60^). When compared with calcium hydroxide, the latter had a significantly more antibacterial effect than bioactive glass in preventing residual bacterial growth. However, the nano-bioactive glass was found to be less effective in eliminating biofilms as compared to the planktonic counterparts ^61^.

Nanoparticles have been employed to improve the overall efficacy of photodynamic therapy by modification of the photosensitizer component ^62^, ^63^. The combination of nanoparticles with photosensitizer enhances the antimicrobial efficacy via several mechanisms: (a) The higher concentration of photosensitizer per mass produces higher yield of reactive oxygen species; (b) The reduced efflux of photosensitizer from bacterial cells decreases the possibility of drug resistance; (c) The ability to rapidly target bacterial cells due to the electrostatic interactions between cationic nanoparticles and bacteria; (d) The greater stability of photosensitizer molecules occurs after conjugation with nanoparticles ^64^. Pagonis et al. ^63^ tested poly (lactic-co-glycolic acid) (PLGA) nanoparticles loaded with the photosensitizer methylene blue (MB) for PDT application. They concluded that cationic MB-loaded PLGA nanoparticles have the potential to be used as carriers of photosensitizer in PDT within root canals. Shrestha and Kishen ^65^ used photosensitizer to functionalize chitosan nanoparticles that possessed the combined properties of chitosan and rose bengal (RB). The combined nanoparticle-photodynamic effect resulted in the complete disruption of multispecies biofilm ^65^. The functionalized chitosan-rose Bengal nanoparticles also demonstrated significantly lower cytotoxic properties. Furthermore, when applied to root canal dentin these nanoparticles crosslinked dentine collagen, which improved the resistance to enzymatic (proteases) degradation and the mechanical characteristics of dentine ^66^. This process of dentine stabilization by combining biopolymeric nanoparticles and crosslinking technique is termed microtissue engineering. A recent study indicated that micro-tissue-engineered root canal dentine enhanced the mechanical characteristics of the root dentin.

In relation to systemic health effects, as nanoparticles are similar in size to biological molecules, they are readily absorbed by various organs and tissues and have been found to accumulate in the lungs, liver, and reticuloendothelial system. Toxic concentrations can cause tissue damage, instigating DNA mutations, cytokine release, protein denaturation, lipid peroxidation, and cell apoptosis. Toxicity reports are mainly associated with AgNPs when compared, for example, with the organic biopolymer chitosan and QPEI nanoparticles ^49^. Nanoparticles can also be associated with environmental effects. They can act as pollutants and accumulate in the environment, and since toxic effects are often concentration-dependent, bioaccumulation can result in subsequent systemic toxicity to exposed living organisms ^49^. Finally, it is necessary to investigate which are the ideal policies for the proper recycling and safe disposal of nanoparticles, since the extent of their harmful effects has not yet been fully elucidated ^49^.

### 3.2 Ozonated Water

The use of ozone in Endodontics has been believed mainly for its powerful bactericidal action and low cytotoxicity ^67^. Additionally, ozone therapy is non-traumatic, painless, and non-invasive, which increases patient acceptability ^68^.

Ozone is a molecule composed of three oxygen atoms with a molecular weight of 47.98g/mol. It is colorless and has a characteristic smell ^69^ (69). It can be used in the forms of oxygen/ozone gas, ozonated water and ozonated olive oil ^25^,^70^,^71^. Ozonated water and oil can retain and release oxygen/ozone; an ideal delivery system. These application forms are used individually or in combination to treat dental diseases ^25^, ^70^. Thermodynamically, a highly unstable gas decomposes into pure oxygen (O₂). It cannot be stored and therefore needs to be used immediately as it has a short half-life of 40 minutes at 20°C and almost 140 min at 0°C ^25^,^69^,^70^.

Oxygen/ozone therapy has a long history of clinical use in humans. In 1839, Christian F. Schonbein noticed the emergence of a pungent gas with an electric smell. Later, in 1857, Wener Von Siemens designed an ozone generator. A few years later, in 1870, Lender made the first medical application, when he purified blood in test tubes. Finally, in 1930, Fisch regularly used ozone in his dental practice in Switzerland ^70^.

Ozone gas has a high oxidation potential and is 1.5 times more effective than chloride when used as an antimicrobial agent against bacteria, viruses, fungi, and protozoa ^25^, ^70^, ^71^. The antibacterial property of ozone occurs not only by damaging bacterial cell membranes by ozonolysis but also by causing oxidation of intracellular proteins, leading to loss of organelle function ^67^. Gram-positive bacteria show more sensitivity to ozone compared to Gram-negative organisms ^68^. Ozone has a selective action against microbial cells and therefore does not affect human cells, resulting in minimal cytotoxicity and high biocompatibility with oral tissues ^67^. In fact, Küçük et al. ^71^ analyzed the cytotoxicity of various concentrations of ozonated water on human primary dental pulp cells. They concluded that ozonated water is non-toxic and induces cell proliferation as well. This proliferation effect was time and dose-dependent.

In addition, it has high efficiency against antibiotic-resistant strains, and its effect increases at acidic pH ^67^. Its other advantages include increased blood circulation, improved cellular immunity and humeral systems, proliferation of immunocompetent cells, immunoglobulin synthesis, macrophage activation, improved wound healing, and lack of mutagenicity ^25^,^67^,^70^.

Regarding endodontic treatment, ozone is considered to be a beneficial choice of antiseptic for the root canal. It is helpful to eliminate not only bacteria but also fungi such as *Candida albicans* ^69^. Ozone can also neutralize toxic endotoxins that irritate the pulp, and thus help the pulp to recover it ^72^. It is possible to use the three forms of presentation during endodontic treatment: the water and gas forms can be used in the rinse protocol, and the oil can be used as intracanal medication ^69^. According to Sen and Sen ^68^, another protocol for use in endodontic treatment would be to have the prepared root canal first lubricated with ozonated oil and then irrigated with ozonated water and dried. Followed by insufflations into each canal should be done with a concentration of ozone gas before root canal filling. The gaseous form provides high penetrability to lateral canals and root deltas, which increases the chance of disinfection ^69^. For a better effect, the amount of organic matter and debris left inside the root canal should be reduced to a minimum, so its use at the end of chemical mechanical preparation is suggested. Finally, although the effectiveness of ozone shows a wide range in many studies, it can be considered as an additional step in the disinfection protocol ^69^.

Despite so many positive properties and numerous research, the effectiveness of ozone as an antimicrobial agent remains very controversial. There are studies favoring its use as a disinfectant agent, as well as studies demonstrating unfavorable results or suggesting it only as a complementary disinfectant ^67^. Silva et al. ^73^ performed a systematic review analyzing whether ozone therapy is comparable to conventional chemo-mechanical techniques using NaOCl about reducing the burden of microorganisms in endodontic treatment. Ozone therapy provides significantly less microbial load reduction than NaOCl. As an adjunct in chemomechanical preparation, ozone was ineffective in increasing the antimicrobial effect of NaOCl. Ozone performance was strongly associated with the application protocol used: it is dose, time, and bacterial strain dependent, in addition to the correlation with the use of complementary disinfection sources.

There is also a need to explore the possible role of ozone in periapical healing and pain control. Because, due to its high oxidative power, there is an increase in adenosine triphosphate synthesis, resulting in improved cellular metabolism and accelerating the repair process by stimulation of angiogenesis ^67^. Pietrzycka and Pawlicka ^74^ clinically evaluated the treatment of infected root canals carried out in one visit, with and without ozone application, or in two visits. The results of the follow-up performed after 6 and 12 months showed that the three methods described showed similar clinical efficiency with a significant decrease in periapical lesions or even complete healing. On the other hand, Silveira et al. ^75^ evaluated, in dogs, the response of the periradicular tissues to the endodontic treatment performed in a single visit or two visits, using different intracanal medications (ozonized oil or calcium hydroxide in camphorated paramonochlorophenol (CMCP). Their results demonstrated that the two-visit treatment offered a higher success rate compared to one-visit therapy. In addition, ozonized oil may potentially be used as an intracanal medication. Regarding pain control, Sinha et al. ^67^ performed a Randomized Clinical Trial comparing the effect of different ozone application techniques on the prevalence of post-endodontic pain (visual analog scale (VAS^)^. The authors concluded that ultrasonic and sonic activation of ozone resulted in less pain in patients compared to treatment without ozone.

There are also studies on the use of ozone gas and water in the treatment of superficial root caries ^25^, ^68^. Al-Omiri et al. ^72^ evaluated post-treatment pain and the need for endodontic treatment after the use of a traditional caries removal method followed by restoration, or after an ozone method of more conservatively managing the deep caries and restoration. Ozone treatment of symptomatic teeth with deep carious lesions almost reaching the pulp shows promise for a more conservative approach to treating deep caries, in addition to being associated with less postoperative pain and less need for endodontic treatment compared to a traditional method.

Ozonated water can also be used as clinical prevention and protection, through pre-dental treatment mouthwash to disinfect the oral cavity. In addition, it can be used as a water supply for the dental chair to prevent the formation of biofilm in the interior and exterior drainage of the chair, as well as it can be used in the ultrasonic unit for prophylaxis. Ozone performs sterilization and leaves only oxygen and water as by-products ^68^.

Other impacts of ozone on the oral cavity include: reduction of halitosis ^68^; helps disinfect gingival and periodontal pockets through irrigation with ozonated water and topical application of ozonated oil to soft tissue ^68^; prevention of dental caries (ozone causes degradation of salivary proteins) ^68^,^72^; reduces dentin hypersensitivity (gas allows diffusion of calcium and phosphorus ions to the deeper layers of dentin through the opening of dentinal tubules) ^68^; ozonated gas can be applied during and after cavity preparations ^68^, ^69^; reduces bacterial adhesion on titanium and zirconia-based implants without altering the adhesion and proliferation of osteoblastic cells ^68^, ^69^; pain control measures, tissue regeneration and healing after tooth extraction using ozonated oil ^68^, ^69^; soft tissue healing in patients with bone necrosis using bisphosphonates; tooth whitening of extrinsic stains (when combined with hydrogen peroxide it has a better effect, resulting in a lighter shade) ^68^; treatment of temporomandibular joint disorders such as trismus, spasm, myoarthropathy; reduction of stomatitis by applying ozone oil on the mucosa under dentures and prostheses ^68^; water or ozonated oil can be applied to soft tissue injuries (herpes, major and minor aphthous stomatitis, removable denture ulcers, angular cheilitis, candidiasis, traumatic wounds, lichen planus, etc.) ^68^; refractory treatment of maxillary and mandibular osteomyelitis together with antibiotic therapy and hyperbaric oxygen therapy ^68^.

However, some of the disadvantages of ozone are its unstable nature in the aqueous state, inconsistent activity on biofilms and Gram-positive bacteria, no residual effect, low diffusivity to deeper areas of the dentinal tubules, and rapid deterioration in the presence of organic tissue ^67^. In addition, there is controversy regarding adverse effects on adhesiveness to enamel and dentin after immediate use of ozone ^70^. According to Rahimi et al. ^25^, ozone appears to not affect dentin adhesion. According to Lubojanski et al. ^69^, ozone can be used to disinfect a surface without affecting the adhesion of pit and fissure sealants. However, according to Küden & Karakaş ^76^, ozone applied on bleached enamel and dentin restricts the bonding of the composite restoration. In addition, ozone inhalation can be toxic to the pulmonary system and other organs ^25^, ^70^. Complications caused by ozone therapy are infrequent up to 0.0007 per application. Known side effects are epiphora, upper respiratory irritation, rhinitis, cough, headache, occasional nausea, vomiting, and shortness of breath, blood vessel swelling, poor circulation, heart problems, and even stroke. Because of ozone's high oxidative power, all materials that are exposed to the gas must be ozone-resistant, such as glass, silicon, and Teflon. However, in the event of ozone intoxication, the patient must be placed in the supine position and treated with vitamin E and N-acetylcysteine ^70^. In addition, ozone therapy is contraindicated in cases of acute alcohol intoxication, recent myocardial infarction, bleeding in any organ, pregnancy, hyperthyroidism, thrombocytopenia, ozone allergy, immunocompromised patients, severe anemia, glucose-6-phosphate dehydrogenase deficiency ^70^ ^) (^ ^70^.

## Activation systems

The effectiveness of irrigation depends on both the mode of distribution and the irrigant properties ^77^. Conventional irrigation depends purely on the positive pressure of injection and the viscosity of the irrigant to flow in the root canal system. Activation in root canal irrigation is the process of employing mechanical, physical, or another form of energy to agitate and improve the flow of irrigants into the intricacies of the root canal system. There are many irrigation activation systems available in the market currently. A systematic review and meta-analysis showed that regardless of the type of irrigation activation technique (device), activation always improves the removal of debris and smear layer ^78^. Therefore, it is an essential step during the chemo-mechanical preparation of the root canal system ^79^. Currently, there are automated systems and manual methods for irrigant activation. Among different activation methods, manual dynamic activation (MDA), passive ultrasonic irrigation (PUI), and sonic irrigation (SI) are some of the most widely used and studied methods ^80^. However, due to the heterogeneity in the techniques and research findings, it is still not possible to recommend any particular technique.

### 4 Conventional Needle Irrigation

Conventional needle irrigation is a widely accepted technique and consists of using an irrigation cannula attached to a syringe. Needles of varying calibers are used, passively or with agitation. The depth of needle penetration and the volume of irrigant can be easily controlled in this technique. However, it is worth mentioning that the fluid flow rate during irrigation is difficult to control and standardize ^79^.

During syringe and needle irrigation, the replenishment and fluid exchange only about 1-1.5mm apical from the tip of the needle irrigation. Generally, the flow of irrigant to the working length and the interaction of the irrigant with the walls of the root canal is dependent on the canal morphology, depth of placement of the needle, diameter of the needle, and position of the needle opening (example- side-vent or open-end) ^81^, ^82^, ^83^. These factors can cause variation in the apical pressure generated during irrigation, which explains some of the rare NaOCl accidents in clinical practice during irrigation with syringe-needle ^84^. Recommendations to avoid NaOCl accidents include: placing the needle tip at an optimum distance from the working length, preventing binding of the needle with the canal wall, and applying a smooth flow rate for irrigant delivery ^85^.

The conventional, syringe-needle irrigation technique often fails to deliver and distribute irrigants effectively within a complex root canal system, especially in the apical third and isthmus areas ^86^. Another challenging factor that limits the effectiveness of the syringe-needle irrigation technique is the so-called vapor lock effect. The vapor lock effect occurs due to the air entrainment in the apical part of the root canal, which prevents the irrigant from reaching the apical portion of the root canal wall ^83^, ^87^. Thus, when syringe-based irrigation is employed with an antibacterial irrigant that substantially reduces the microbial loads in the root canal lumen but the bacteria in the dentinal tubules often remain unchanged. This may negatively affect the prognosis of root canal treatment and in some cases may contribute to persistent apical periodontitis ^80^.

### 5 Manual Dynamic Activation (MDA)

Manual dynamic activation (MDA) is a low-cost technique that does not require additional equipment. This activation system consists of repeatedly inserting a well-fitted gutta-percha cone, hand files, and brushes, adjusted to the working length in an instrumented canal, using short longitudinal push / pull strokes of 2 mm amplitude at a frequency of 100 strokes in about 1 min. This process produces a hydrodynamic effect by displacing/exchanging the irrigant through the root canal system while aiding better interaction with the canal wall (88). This technique is capable of removing the apical vapor lock, improving the debridement, cleaning, and antimicrobial action of irrigants ^77^,^78^,^79^,^80^,^85^.

### 6 Passive Ultrasonic Irrigation (PUI)

**Figure 1:**
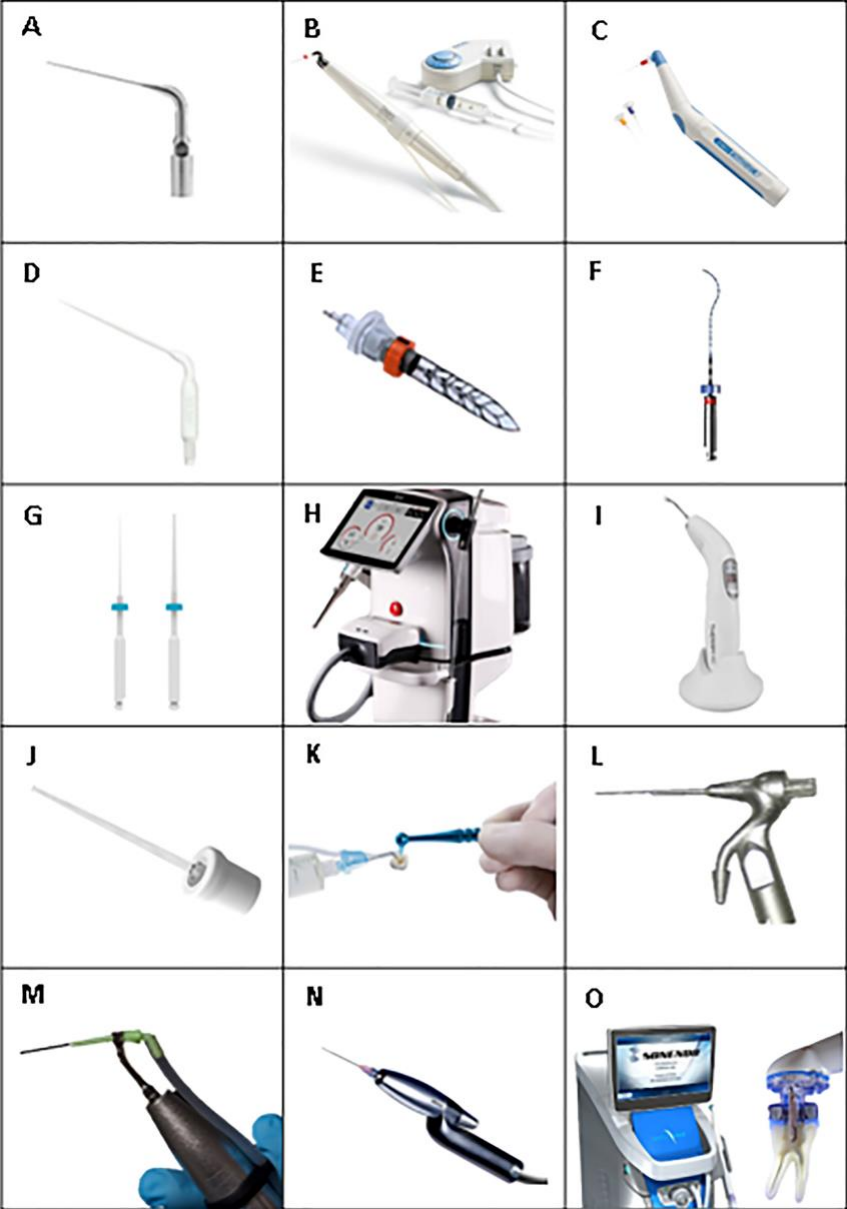
(A) Irrisonic (Helse Dental Technology)*, (B) Continuous ultrasonic irrigation (CUI) cannula (ProUltra PiezoFlow [Dentsply Sirona])*, (C) Endoactivator (Dentsply Sirona)*, (D) EDDY tip (VDW)*, (E) Self-Adjusting File (SAF) file (ReDent-Nova)*, (F) XP Endo Finisher (FKG Dentaire)*, (G) EasyClean tip (Easy Dental Equipment)*, (H) Dental Er:YAG used for PIPS and SWEEPS (Fotona)*, (I) Wireless Therapeutic Laser Equipment (DMC ABC Equipment)*, (J) PDT light guide (DMC ABC Equipment)*, (K) Endovac System (Discus Dental)*, (L) Prototype of CANUI**, (M) iVac system (Pac-dent), (N) RinsEndo device (Dürr Dental)*, (O) Gentlewave irrigation system (Sonendo)*

*Source: figure obtained from the manufacturer's website

**Source: figure obtained from the article doi: 10.1038/s41598-021-90430-0

Passive ultrasonic irrigation (PUI) is one of the most widely used automated irrigation methods. The term "passive" in PUI is a misnomer since it relates to the 'noncutting' action of the ultrasonically agitating tip, but in fact, it has an active action ^22^. This method of irrigation is employed following root canal preparation and enlargement. During the ultrasonic activation process, a small diameter non-cutting metal insert is placed in the root canal and must vibrate freely to transmit energy from the tip to the irrigant. In this case, the ultrasonic oscillation frequency of 25-40kHz is achieved with either magnetostrictive or piezoelectric devices (i.e. Irrisonic tip [Helse Dental Technology, Santa Rosa de Viterbo, SP, Brazil] - Figure 1A). The tip or insert is positioned inside the canal close to the apical region (around 2 mm short of working length) without touching the dentinal walls ^80^ ^) (^ ^70^ ^) (^ 82 ^) (^ 86. It is suggested that for ultrasonically-assisted irrigation to be effective, the tip must operate within a space that is 3 times larger than its diameter ^90^.

Ultrasonic agitation promotes acoustic streaming (or microstreaming) and hydrodynamic cavitation to enhance root canal cleaning efficacy. Acoustic streaming is the rapid movement of fluid in a circular motion around the agitating tip. The multiple nodes and antinodes under the high-frequency of oscillation induce intense acoustic microstreaming. Cavitation is the impulsive formation of cavities in liquid through pressure gradients. Two types of cavitation bubbles occur for ultrasonic activation: Non-inertial and inertial cavitation bubbles. Non-inertial bubbles undergo linear pulsation after exposure to a low-amplitude ultrasonic agitation. Inertial bubbles undergo high-energy pulsations and their collapse generation power shock waves ^91^. In root canals, the effect of non-inertial bubbles and acoustic microstreaming is more significant. Inertial bubbles and cavitation may minimally occur restricted to the tip at high energy ^91^,^92^.

The ultrasonic activation of 1 minute for each canal, with 3 cycles of 10-20 seconds (each with irrigation renewal) has been considered ideal for obtaining clean canals ^40^. A shorter activation time facilitates the maintenance of the tip in the canal center, preventing it from touching the walls ^82^. However, the effectiveness of the PUI is highly dependent on the power intensity of the device, the free space within the canal, and the total absence of interference at the tip. In addition, because of the anatomical characteristics of the root canal, ultrasonic activation is less effective in the apical region than in the cervical region ^93^. Furthermore, uncontrolled dentin removal with PUI, resulting from file-to-wall contact in apical third even within the manufacturer-recommended power settings and a canal enlargement to size # 35/.06 or .04 ^94^,^95^.

During PUI, two flushing methods can be used, continuous or intermittent flush of the irrigant.

### 7. Continuous Ultrasonic Irrigation (CUI)

Continuous ultrasonic irrigation (CUI) provides an uninterrupted supply of fresh irrigation solution in the root canal, improving the physical removal of surface adherent biofilm bacteria and reducing the time required for ultrasonic irrigation. Continuous ultrasonic irrigation (CUI) is based on the activation of an insert connected directly to the ultrasonic unit, which allows a continuous delivery of the irrigant and simultaneous activation of the insert within the root canal (Figure 1B). The irrigating solution passes through the insert in an activated state, allowing the placement of the insert to about 75% of the working length ^89^. CUI also promotes the physical phenomena of microacoustic current and cavitation. However, it has been demonstrated that CUI generated consistently high fluid velocity and shear stress through the apical 3 mm resulting in improved physical removal of surface adherent biofilm bacteria ^96^.

### 8. Intermittent Ultrasonic Irrigation (IUI)

In intermittent ultrasonic irrigation (IUI) the irrigant is injected into the root canal with a syringe, the irrigant solution is then activated with an oscillating ultrasonic instrument and the canal is filled several times after each activation cycle. The depth of penetration of the syringe and the volume of irrigant can control the amount of irrigant flushed through the apical region of the canal. Therefore, the amount of irrigant activated in IUI is small, which also contributes to its limited debridement efficacy in comparison with methods that provide continuous root canal irrigation, replacement, and activation ^97^.

### 9. Sonic Irrigation (SI)

Sonic irrigation differs from ultrasonic irrigation in that it operates at a lower frequency (1-10kHz). It consequently produces lower fluid velocity and shear wall stresses. Sonic activation, on the other hand, generates significantly greater amplitude (horizontal tip displacement) ^80^, ^98^. The oscillation patterns of sonic devices are different when compared to the ultrasonically assisted instruments. A minimum amplitude of oscillation represented a node, while a maximum amplitude of oscillation represented an antinode. Sonically activated tips have one node near the attachment (device) of the tip and one antinode at the free-end of the tip. When the sonic movement of the file is restricted, the lateral oscillation disappears. This results in a vibration purely in the longitudinal direction. This mode of vibration is particularly efficient for root canal debridement because it is largely unaffected by loading and exhibits large displacement amplitudes ^85^. The generation of oscillatory fluid dynamics by contemporary sonic irrigation devices can be achieved using metallic files (for example, Ripsisonic and Shapersonic files, Micro-Mega, Besancon, Cedex, France), conventional ventilated needle tips (for example, Vibringe, Cavex Holland BV, Haarlem, Netherlands) or disposable polymer tips (e.g. Endoactivator, Dentsply Sirona, Ballaigues, Switzerland; and EDDY, VDW, Munich, Germany) ^40^.

### 9.1 Endoactivator

The EndoActivator system (Dentsply Sirona, Ballaigues, Switzerland) is a portable, wireless handpiece, composed of a highly flexible polymer tip that oscillates at frequencies of 1-10kHz ^79^, ^85^, ^99^ (Figure 1C). These disposable tips have three different diameters (15/02, 25/04 and 35/04). They are smooth and do not cut root dentin. The tip design allows safe activation and vigorous agitation of the intracanal fluid. Horizontal agitation of the tip, in combination with short vertical strokes, synergistically produces a powerful hydrodynamic phenomenon inside the root canal. It improves lateral penetration, circulation, and flow of irrigants into the inaccessible locations of the root canals, making cleaning more effective ^85^,^100^,^101^. A possible disadvantage of the polymer tips used in the EndoActivator system is that they are radiotransparent. Although these tips are disposable and do not break easily during use, it would be difficult to identify them if part of a tip is separated within a canal ^85^.

### 9.2. EDDY

The EDDY system (VDW, Munich, Germany) is a passive sonic irrigation system made of flexible polyamide in order to avoid cutting the dentin and altering the canal morphology (Figure 1D). The device is non-cutting, sterilized, disposable, and is activated at 5000 to 6000Hz by an air-driven handpiece (Air Scaler). The vibration produced is transferred to the tip, which moves in a high-amplitude oscillatory movement. This three-dimensional movement triggers “cavitation” and “acoustic transmission”. According to the manufacturer, this system allows both an efficient cleaning of the complex root canal system and the removal of debris and organic tissues ^99^,^102^.

### 10. Special Endodontic files.

Special endodontic files have been developed to improve the effectiveness of irrigation and disinfection during chemomechanical preparation, including SAF (Self Adjusting file) and XP Endo Finisher, among others.

### 10.1. Self-Adjusting File (SAF)

The Self-Adjusting File (SAF) system (SAF, ReDent-Nova, Ra’anana, Israel) consists of a self-adjusting file (SAF) operated with a special RDT handpiece head and an irrigation pump (either the VATEA pump or the all-in-one EndoStation unit) that delivers a continuous flow of irrigant through the hollow file (Figure 1E). Because the file is built as a lattice-walled cylinder, no pressure is generated within the file; any small pressure that is generated by the pump to deliver the irrigant through the tube is eliminated the moment the irrigant enters the file ^103^.

SAF was introduced with the concept of a single instrument to prepare the entire root canal. It consists of a hollow file designed with two parallel longitudinal beams of thin-walled compressible (1.5 mm) pointed cylinder. The longitudinal beams are held together by Ni-Ti lattice, which are 120 μm thick with an abrasive surface. During operation, the SAF adapts three-dimensionally to the irregular shape of the root canal, applying constant and delicate pressure to the canal walls, which helpsto reduce the incidence of dentinal microdefects. Instead of instrumenting the root canal into a round cross-section, the SAF maintains the original shape of the canal with slightly larger dimensions ^104^, ^105^, ^106^. The hollow design helps in the continuous flow of endodontic irrigants throughout the procedure and they are activated by sonic stirring ^104^, ^106^. Vertical vibrations delivered by SAF ensures good debridement and disinfection by a scrubbing action as the file adapts well to the canal walls ^77^.

According to Metzger ^103^, the RDT handpiece-head has a dual mechanical function. It turns the rotation of the micro-motor into a trans-line in-and-out vibration with an amplitude of 0.4 mm. It also contains a clutch mechanism that allows the SAF to rotate slowly when not engaged in the canal but completely stops the rotation once the file is engaged with the canal walls. The micromotor is operated at 5000 rpm, which results in 5000 vibrations/min, and the operator uses pecking motions when using the SAF. Free rotation of the file should occur at every out-bound part of every pecking stroke, when the SAF file is disengaged from the canal walls. This is required to ensure that when the SAF enters the canal during the in-bound pecking motion, it will do so at a different, random circular position every time, thus ensuring uniform treatment of the canal walls. This random circular position also allows the asymmetrical tip of the file to negotiate curvatures that may be found in the root canal. RDT heads are available in several configurations and may be adapted to a large variety of endodontic motors/handpieces.

The VATEA (ReDent) is a self-contained peristaltic pump with a built-in irrigant reservoir of 500 mL operated using a foot switch and powered by a rechargeable battery. The SAF file is provided with a freely rotating hub connected to a polyethylene tube, thus allowing for flow of the irrigant through the hollow file and into the root canal. The irrigant can be delivered into the tube at a rate ranging from 1-10 mL per minute, with the typical recommended setting of 4 mL per minute ^103^.

The EndoStation, an all-in-one endodontic unit (ReDent and Acteon) is a compound machine specifically designed for the SAF that uses a special RDT handpiece. Nevertheless, it can also be operated using a conventional handpiece with either rotary or reciprocating files. The EndoStation is equipped with a peristaltic pump that enables continuous irrigation when used in "SAF mode". An external bottle is used as the irrigant container of the EndoStation, from which the irrigant is drawn by the peristaltic pump into the tube and through it to the attached file. When used in "SAF mode", both the micromotor and the irrigation pump are simultaneously operated using a single foot pedal ^103^.

The SAF System may be defined as a no-pressure irrigation system that is applied throughout the instrumentation process. Once the irrigant enters the SAF, any pressure that may have existed in the delivery tube disappears due to the lattice structure of the file. The irrigant is continuously delivered into the root canal, and the vibrations of the file combined with the pecking motion applied by the operator result in the continuous mixing of the irrigant that is present in the root canal with fresh, fully active irrigant ^103^.

Metzger et al. ^107^ evaluated the quality of root canal preparation and root canal obturation in canals treated with either rotary or SAF, using three-dimensional micro-computed tomographic (CT) analysis. The SAF allowed better cleaning and shaping and better adaptation of the root canal filling than those allowed by rotary files.

A study comparing the efficacy of the SAF system with continuous NaOCl irrigation, against the ProTaper rotary file system plus syringe-based irrigation (NaOCl) on the debris, smear layer removal, and presence of bacteria showed no significant difference between these two techniques on the degree of microbial reduction in the root canal lumen. Conversely, higher bacterial reduction was observed in dentin shaving obtained from the ProTaper rotary file system. It was concluded that the SAF system does not allow control of apical instrumentation or enlargement, thus limiting the ability of the irrigant to achieve effective and predictable disinfection ^108^.

The concept of a 3D file that adapts to the root canal morphology is an excellent approach. However, the degree of microbial reduction with the SAF needs further investigation.

### 10.2 XP Endo finisher

XP Endo Finisher (FKG Dentaire, La Chaux-des-Fonds, Switzerland) is a non-tapered nickel-titanium (NiTi) instrument of size #25 (Figure 1F). The NiTi alloy in this instrument is thermomechanically treated and is termed MaxWire (Martensite-Austenite-Electropolish-Flex). These instruments are relatively straight in their M phase (martensitic state) at room temperature ^109^. The treated alloy changes from the martensitic to austenitic phase at temperatures equal to or greater than 35°C ^110^ and this change gives the file a spoon shape with a depth of 1.5 mm for 10 mm of its length, formed by the molecular memory ^111^, which performs the eccentric rotational movement ^110^.

The recommended operating speed with irrigation solutions is 800 rpm ^111^ and 1 Ncm in slow up-and-down movements ^109^. XP Endo Finisher should be used after preparing the root canal for size #25 or greater ^111^.

The austenitic phase transformation allows the instrument to expand its length by 6 mm in diameter when rotated ^109^. This file system contributes to the removal of smear layer, debris, medication, biofilms, and filling materials from the root canal system ^110^.

Recently, another file of this system was developed, the XP-Endo Finisher Retreatment (XP - Endo Finisher R), which can also expand at body temperature, taking the shape of a snake ^112^. The XP-Endo Finisher R file has a slightly larger diameter, size 30, and does not have a taper ^110^. This new file aims to improve cleanliness during root canal retreatment ^110^,^112^.

Carvalho et al. ^113^ observed that the use of the XP-endo Finisher as a supplementary approach to the irrigation/instrumentation technique improved the cleaning efficiency of root canals of both tested file systems (XP-endo Shaper and Reciproc Blue) and irrigating substances (0.9% NaCl and 2.5% NaOCl).

### 11. Polymer device

### 11.1 EasyClean

The EasyClean system (Easy Dental Equipment, Belo Horizonte, MG, Brazil [U.S. patent pending 61 / 849,608]) is an acrylonitrile-butadiene-styrene (ABS) polymer device of size #25 .04 taper (Figure 1G). It has a cross-section in the shape of an airplane wing that operates in a reciprocal movement (180° in a clockwise direction and 90° in a counterclockwise direction) or a continuous rotation movement. The basic principle of the EasyClean system is the mechanical agitation of the chemical irrigant, facilitating mechanical dislodgment of the debris and smear later adhering to the canal walls. As it is a flexible polymer instrument of a small caliber, its performance is affected neither by the root canal space nor by the contact with the root canal walls. It can be introduced up to the working length, which optimizes the action of irrigating solution in the uninstrumented portions of the root canal ^89^,^93^,^100^,^111^.

Aveiro et al. ^28^ observed that Easyclean in reciprocating movement did not cause a statistical difference in relation to the reduction in microbial load within root canals when compared to conventional irrigation. Moreover, Duque et al. ^100^ demonstrated that Easyclean used in continuous rotation provided better cleaning of the canal and isthmus than conventional irrigation. This probably happened because of the difference in rotational speed that produced turbulence of the irrigating solution, favoring debris removal from the isthmus.

### 12. Light-based Adjunct Therapy

According to Meire et al. ^114^, a popular form of adjunct therapy is represented by the use of light. The first approach is the use of laser light for direct canal wall irradiation, where the canal walls are exposed to irradiation with laser light of a particular wavelength, typically in the absence of an irrigant. Mostly, near-infrared laser light is used for this purpose, for example, 980-nm wavelengths diode laser light/ or 1064-nm neodymium-doped yttrium aluminum garnet (Nd:YAG) lasers.

Another form of light-based adjunct therapy is the use of laser light to activate root canal irrigants, called laser-activated irrigation (LAI). In this approach, pulsed laser light is targeting the irrigant within the root canal, to improve irrigant dynamics, distribution, and cleaning action. For this purpose, far-infrared laser light including 2780-nm /2790nm erbium chromium, yttrium scandium gallium garnet (Er;Cr:YSGG laser) ^115^ / or 2940-nm erbium-doped yttrium aluminum garnet (Er:YAG) ^116^, ^117^ are typically used.

Antimicrobial photodynamic therapy (aPDT) represents a different light-based adjunct therapy. It is the intracanal application of a photosensitizer (compound selectively binding microbial cells), followed by irradiation by light whose wavelength matches the absorption peak of the photosensitizer, resulting in a chemical reaction that produces microbicidal elements.

### 12.1 Laser activation using Er:YAG laser

### 12.1.1 Photon-induced photoacoustic streaming (PIPS)

PIPS is a laser activation technique that activates irrigant solutions commonly used in endodontics with low-energy laser (Erbium:YAG laser-Er: YAG) (Figure 1H) that emits an infrared light with a wavelength of 2940 nm. PIPS uses a tapered, stripped, radial firing tip with laser pulses of subablative energies of 20 mJ at 15 Hz for an average power of 0.3W at super short pulses of 50 µs. These impulses induce the interaction of water molecules with peak powers of 400W ^116^. A laser wavelength that has water as its corresponding chromophore offers the best chance for optimal results, since water is the vehicle for irrigants, and therefore minimizes the thermal component arising from the laser-tissue interaction ^118^.

When an Er: YAG laser is fired in an aqueous medium, the irrigant is locally and instantly heated beyond its boiling point and a vapor bubble begins to form at the end of the fiber tip after each pulse. This vapor bubble collapses after reaching its maximum volume with a subsequent cavitation effect. This phenomenon produces turbulent photoacoustic agitation of irrigants that flow fluids three-dimensionally throughout the root canal system and leads to the effective removal of the smear layer ^119^. In addition, the Er: YAG laser also dissociates water molecules generating hydroxyl ions that can further potentiate the antimicrobial effect ^118^. Compared with conventional irrigation, this technique has the positive effect of allowing better penetration of irrigant into the dentinal tubules, which leads to a significant improvement in the removal of smear layer, debris, intracanal medicatio,n or bacteria from the root canal walls ^120^.

Do and Gaudin ^121^ conducted a literature review about the efficiency of the Er: YAG laser and PIPS, and unfortunately, there are no clear recommendations in the literature about irrigation or activation times. The duration of the application must be as short as possible, but with maximum efficiency. The authors observed a wide range of activation times from 20 seconds to 240 seconds with no consensus.

However, its efficacy in achieving bacterial reduction in the root canals still needs more investigation ^40^.

### 12.1.2. SWEEPS (Shock Wave Enhanced Emission Photoacoustic Streaming)

SWEEPS is a technique that places a laser fiber tip in the access cavity filled with irrigants and emits a pulsed laser light into the fluid (Figure 1H). It is a more recent Er: YAG laser modality launched to improve the cleaning and disinfection efficiency of the PIPS technique. PIPS triggers a single laser pulse with a square waveform in each emission cycle. In contrast, SWEEPS uses synchronized pairs of ultrashort pulses over an ideal time interval to accelerate the collapse of laser-induced bubbles. This characteristic results in increased shock wave emission and fluid dynamics, even within the narrowest portions of the root canal ^117^,^119^,^122^.

However, for SWEEPS, there are fewer studies investigating the characteristics of the irrigant flow and efficacy of SWEEPS-based irrigation on complex root canal morphology and bacterial biofilms ^122^.

### 12.2. Antimicrobial photodynamic therapy

Antimicrobial Photodynamic therapy (aPDT) employs a light-sensitive chemical (photosensitizer) at extremely low and non-toxic concentration, which when activated with low-level light, produces reactive oxygen radicals that causes bacterial killing ^123^. The wavelength of light in aPDT should correspond with the absorption wavelength of the photosensitizer. The photosensitizer in the triplet state is extremely reactive and is capable of destroying bacteria. Previous studies have shown that the photo-oxidative effect caused by photosensitizer in bacteria caused damage of multiple targets in bacterium such as membrane integrity, protease activity and chromosomal DNA ^124^. The selectivity of photodynamic effect towards microbial elimination over eukaryotic cells is an important advantage with aPDT ^125^,^126^. These findings support the hypothesis that aPDT for root canal disinfection and is considered a feasible alternative to antibiotics ^127^, ^128^.

Clinically, aPDT involves two steps. In the first step, the photosensitizer solution is applied within the root canal using a syringe-needle (30G) (photosensitization period). The photosensitization period may last from 1-5 min. The photosensitizer in this period is expected to bind to bacteria. In the second step, the excess photosensitizer is removed from the canal and light illumination is carried out for appropriate dose using a fiberoptic delivery system (Figure 1I e 1J). Light sources used for aPDT can be coherent (lasers) or non-coherent (lamps) ^123^. Lasers provide a monochromatic, coherent, and collimated light with a wide range of output power. Nd:YAG, HeNe, GaAlAs and diode lasers, light emitting diodes (LEDs), and xenon-arc lamps have been used for aPDT ^123^,^129^. Light from any source can be easily coupled into a fiber optic that can serve as a delivery probe for application in endodontic treatment. The phenothiazinium group of photosensitizers such as Methylene blue and TBO have been commonly used for clinical application ^130^. Other photosensitizers such as porphyrins, phthalocyanines, chlorins, rose bengal, and erythrosine have also found application in aPDT.

Some of the tissue-specific challenges for aPDT in endodontic disinfection are the penetration of optimum light energy into the root canal/dentin, diffusion of photosensitizer into the complexities of the root canal and limited availability of environmental oxygen in the infected tissue, which could impede the singlet oxygen species produced by aPDT ^123^. The presence of organic tissue remnants, dentin powder, and serum may also compromise the antimicrobial efficacy of aPDT. This may be due to cross-linking action of singlet oxygen, compromised half-life of singlet oxygen, or non-specific binding of photosensitizer ^131^.

In an approach to improve the antimicrobial efficacy of aPDT in endodontic applications, methylene blue was dissolved in different formulations. One such formulation containing glycerol and ethanol was found to effectively penetrate dentinal tubules, enhance singlet oxygen generation, and improve bactericidal action inside the root canal ^132^. A significantly higher impairment of bacterial cell walls and extensive damage to chromosomal DNA was also observed with this improved photosensitizer formulation. Along the same line, the incorporation of an oxidizer and oxygen carrier with photosensitizer formulation in the form of an emulsion was also shown to produce significant photooxidation capabilities, which in turn facilitated the comprehensive disruption of endodontic biofilms ^124^. Modification of photosensitizer by conjugating with other chemical moieties can result in improved photosensitizers with significantly high reactive oxygen release for A-PDT. In one such attempt, covalently conjugating a photosensitizer to chlorin (e6) and a poly-l-lysine chain was found to enhance antibacterial efficacy ^133^. Photosensitizer conjugated with positively charged chitosan has also been shown to be highly effective in removing biofilms of gram-positive and gram-negative bacteria ^134^.

Systematic reviews have concluded that aPDT reduced bacterial counts in most in vitro studies, especially when used as an adjunct to the conventional endodontic technique to treat refractory infection. However, limited clinical information is currently available on the use of aPDT in root canal disinfection. If supported by future clinical research, aPDT may have efficacy for additional root canal disinfection, especially in the presence of multi-drug-resistant bacteria ^135^,^136^.

Further application of aPDT includes biostimulation, attenuation of inflammation, induction of bone regeneration, and analgesic properties ^29^,^137^,^138^,^139^.

### 13. Apical Negative Pressure Irrigation (ANPI)

According to Konstantinidi et al. ^140^ in a systematic review on apical negative pressure irrigation versus syringe irrigation, negative pressure irrigation is an alternative method for the delivery of irrigants inside the root canal that was proposed to minimize the risk of irrigant extrusion through the apical foramen. Irrigants are delivered by a syringe and needle inside the pulp chamber and a fine suction tip placed near the working length creates the necessary negative pressure that drives the irrigant into the canal. Several studies have compared this method to syringe irrigation and it appears that negative pressure irrigation can indeed prevent irrigant extrusion through the apical foramen in vitro. However, there is insufficient evidence to claim the general superiority of any one of these methods.

### 13.1 Endovac System

The Endovac system (Discus Dental, Culver City, CA) is a negative pressure irrigation device that was designed to deliver the irrigant (NaOCl) at the apical portion of the root canal systems and to suck by negative pressure the debris from the root canals (Figure 1K). This system consists of a Master Delivery tip, a macrocannula, and a microcannula that are connected to a vacuum line. Using this system, irrigants are delivered at the pulp chamber with the Master delivery tip ^101^. The irrigant is drawn through the canal walls towards the root canal working length, by the negative pressure applied by the microcannula. This mechanism helps to avoid vapor lock, thus allowing effective irrigation, and has the ability to safely supply irrigant up to the working length without causing irrigant extrusion at the periapical region ^78^,^85^,^98^.

In the EndoVac system, a macrocannula or microcannula is connected via a tube to an irrigation syringe and the high-speed suction of a dental unit. During irrigation, the delivery/evacuation tip (Master Delivery Tip) supplies the irrigation to the pulp chamber and siphons the excess irrigation to prevent overflow. The cannula in the canal simultaneously exerts a negative pressure that pulls the irrigator from its new supply into the chamber, descends through the canal to the tip of the cannula, into the cannula, and out through the suction hose. Thus, a steady stream of fresh irrigators is being supplied by negative pressure to the working length. The plastic macrocannula has an open end measuring 0.55mm in diameter, with a 0.02 taper, is attached to a titanium loop, and is used for initial gross cleaning of the coronary part of the root canal. The stainless steel microcannula measures 0.32 mm in diameter and has 12 laser cut perforations (4 sets of 3 holes), positioned laterally, adjacent to its closed end (85). This is attached to a titanium finger-piece for irrigation of the apical part of the root canal. In order to position the microcannula at the working length, the root canal is prepared to a minimum size #35 ^85^ ^) (^ ^104^.

### 13.2 CANUI (Continuous Apical Negative-Pressure Ultrasonic Irrigation)

The use of ultrasonic systems is one of the alternatives to clean root canal systems and improve disinfection. However, this method can transport the irrigant further than the distance the instrument acts, compromising the safety of the procedure with the extrusion of NaOCl in the periapical tissues. On the other hand, the literature shows the absolute safety of negative pressure cleaning systems compared to syringe irrigation and ultrasonic irrigation. In addition, in irrigation of curved canals, ultrasonic irrigation can cause irregularities in the preparation ^141^.

Continuous apical negative-pressure ultrasonic irrigation (CANUI) makes use of a new device for activating the irrigant in a root canal system with an ultrasonic dental unit (Figure 1L). The advantage of this technique is the combination of negative apical pressure, which avoids apical extrusion of the irrigants, together with continuous ultrasonic irrigation, where there is constant renewal of the irrigant, which optimizes its penetration into the ramifications of the canal ^141^.

The device consists of a tube inside another tube that allows the continuous ultrasonic exchange of fresh irrigant, as the irrigant is simultaneously aspirated apically. The coronal and apical tubes are 0.75 and 0.3 mm in diameter, respectively. It is composed of a nickel-titanium microcannula suitable for the working length of curved canals. The device is mounted on an ultrasonic unit, with the power set to level 6 (equivalent to an approximate frequency of 25 kHz). A 10 ml syringe containing the irrigant is attached via a tube. The microcannula is connected to the suction part of the dental unit to aspirate the irrigant ^141^.

During instrumentation, the CANUI device is inserted into the coronal and middle portions of the canal to clean and disinfect the root canal system. After instrumentation is complete (it is necessary instrument to be at least 30.06), the CANUI is inserted until the apical end of the microcannula reaches a point 0.5 mm shorter than the working length. The inactive device is placed in the canal and then the release of the solution is initiated. At this point, the device can reach its full cleaning potential ^141^.

Castelo-Baz et al. ^141^ evaluated the efficacy of continuous apical negative ultrasonic irrigation in simulated lateral canals and the apical third in straight and curved root canals. CANUI improves penetration into the lateral canals and up to the working length of the cleared teeth in straight and curved roots.

### 13.3 iVac™ Apical Negative Pressure Irrigation and Activation System

iVac (Pac-dent, Brea, CA, USA) combines the ultrasonic activation of the irrigant, which optimizes the penetration of the irrigant in the areas of anatomical complexities, with a negative apical pressure system, which prevents the apical extrusion of the irrigant even if used at working length. In addition, this device uses a flexible tip that allows its use in curved root canals and makes the chance of cannula separation extremely low, if there is any chance ^142^ (Figure 1M). iVac microcannula is composed of a polymer, which allows effective ultrasonic activation of the irrigant, even in curved canals, while the CANUI system, is composed of a nickel-titanium microcannula ^141^.

The iVac system is simple to install and has an intuitive operation, requiring no significant equipment investment. In addition to the ultrasonic vibration, the cannula's external and internal diameter rate significantly reduces the risk of clogging ^142^.

The kit contains an aspiration/activation polymer cannula with two options of outside diameters, .35mm (green tip) and .50mm (yellow tip). The cannula is attached to an ultrasonic piezo connector, and the connector is coupled to a piezoelectric ultrasonic handpiece. In this way, there will be vibration in the cannula and concomitant irrigation from the reservoir of the ultrasonic piezo unit. The iVac piezo ultrasonic connector provides a continuous flow of irrigant, projecting the liquid onto the outer surface of the cannula. The vibration will help carry the irrigant along the entire length of the canal and then it will be collected through the apical opening of the cannula. The other end of the cannula will be connected to the standard evacuation tube, creating negative pressure fed by the equipment. Furthermore, the cannula is disposable and the ultrasonic connector can be cleaned, sterilized, and reused. The iVac system can be used with most ultrasonic piezo units on the market ^142^.

Preliminary findings of our laboratory evaluated the effectiveness of conventional irrigation, ultrasonic activation and activation with the iVac system using 2.5% NaOCl and saline solution, in the reduction of a multispecies biofilm in the root canal and intratubular dentin, reduction of lipopolysaccharides and evaluated the apical extrusion using different root canal instrumentation thresholds (0 and -1mm). The iVac group stood out when analyzing the reduction of CFU in intratubular dentin and in relation to the apical extrusion of irrigants regardless of the instrumentation limit used.

### 9. Hydrodynamic pressure (positive pressure)

The RinsEndo® device (Dürr Dental GmbH, Bietigheim-Bissingen, Germany), is a mechanism for hydrodynamic root canal irrigation that combines simultaneous irrigation and aspiration under hydrodynamic pressure (positive pressure) and activates it automatically. The system is connected directly to the turbine cable, and the irrigating liquid is pumped through cannulas, with 30 gauges in diameter and 7 mm lateral opening, into the canal, with pressure from 2 to 5 bar, with a volume of 6.2 ml/min with a frequency of 1.6 Hz ^143^,^144^ (Figure 1N).

The RinsEndo® has been shown to be superior over conventional syringe/needle irrigation in terms of deeper penetration of an irrigant in dentine, and reduction of the number of bacteria 143 . Hydrodynamic activation improves the circulation and flow of the irrigant into the difficult-to-access areas of the root canal system and promotes its dentin penetration ^145^. However, the comparison with PUI yielded contradictory results ^143^.

The manufacturer’s instruction suggests that the apical third of the root canal is effectively irrigated although the needle tip is inserted just into the coronal third because of the hydrodynamic activation of the irrigant, which was confirmed by Rödig et al. ^146^. The device removed significantly more debris from the apical root canal irregularities when the needle tip was placed the most coronally. However, passive ultrasonic irrigation was more effective than syringe irrigation or RinsEndo in removing debris from artificial extensions in straight root canals whereas the size of the apical preparation does not play a decisive role ^146^. On the other hand, in a laboratory study, Magni et al. ^147^ determined that performing irrigation with RinsEndo or EDDY in teeth with an open apex produced pressures higher than the critical threshold ^147^. Hauser et al. ^148^ also proved in a laboratory study that the hydrodynamic rinsing enhanced the penetration depth into root canal wall dentine. However, apical extrusion of NaOCl was a common occurrence.

### 15. Gentlewave irrigation system

The Gentlewave irrigation system (Sonendo, Inc., Laguna Hills, CA, USA) is a device developed for cleaning and disinfecting a minimally instrumented root canal 149 (Figure 1O). This system uses NaOCl and EDTA, with a rinse of distilled water between them. It applies a Multisonic technology, which means that multiple acoustic frequencies are generated at the same time, and when this technology is paired with optimized procedure fluids, it brings about cleaning and disinfection of the entire root canal system, regardless of any anatomical complexities ^150^,^151^,^152^.

Studies suggest its effectiveness in several stages of endodontic treatment: dissolving soft tissues eight and ten times faster than ultrasonic devices and needle irrigation, respectively ^84^; calcium hydroxide removal ^153^; removal of filling material ^102^; removal of calcifications ^154^ and even the removal of fractured manual files in the apical (61%) and middle (83%) thirds without the need for further wear on the dentinal structure ^155^. In addition, in a multicenter clinical study, Sigurdsson et al. ^151^, reported a 97% success rate for teeth treated with the Gentlewave system after 12 months of follow-up.

The GentleWave system consists of a console and a sterile single-use handpiece. During treatment, the tip of the handpiece is kept inside the pulp chamber, approximately 1 mm above the floor of the pulp chamber, without the tip entering the root canals, thus allowing minimal instrumentation and saving integrity and tooth strength. During use, the pulp chamber of the tooth is sealed from the oral cavity, preventing the mist of NaOCl from spreading through the work field. A stream of treatment solution is supplied from the tip of the handpiece to the pulp chamber at approximately 45 mL/min. Excess fluid, as well as debris and dissolved tissue from the root canals as well as the pulp chamber, are simultaneously removed through small suction holes in the handpiece sealing cap to a waste container inside the console. The current of the treatment fluid interacts with the stationary/stagnant fluid within the pulp chamber creating a strong shear force, which causes hydrodynamic cavitation in the form of a cavitation cloud (microbubble implosion). This strong cloud of hydrodynamic cavitation generates a wide spectrum of sound waves within the degassed fluid. Degassed fluid refers to the treatment fluid with a reduced amount of dissolved gas to optimize the distribution of this energized fluid (i.e., multisonic energy and fluid dynamics) throughout the root canal system. Multisonic energy (energy generated by various wavelengths of sound over a wide range of frequencies) travels through the fluid to the entire root canal system. The treatment tip of the handpiece is designed to deflect the flow of treatment fluid in order to generate a flow over the orifices of the root canals. This flow induces a smooth vortical flow, which creates a slight negative pressure within the root canal system. Energy and vortical flow dissipate as they travel apically to the root canal system. The interaction of multisonic energy, the dynamics of the vortical fluid, and the chemistry of the treatment fluid result in improved dissolution and removal of organic matter, that is, pulp tissue and biofilm from the root canal system ^84^,^149^,^150^,^151^,^152^,^156^. An in vitro study investigated the effectiveness of ultrasonic (PiezoFlow system) and Gentlewave system in removing multispecies oral biofilms from root canals. The root canals in the ultrasonic group were prepared to size #35/.04, while in Gentlewave group was prepared to size #15/.02. It was reported that both the tested systems demonstrated similar reduction in microbial load, although the number of residual bacterial DNA was significantly smaller in the GentleWave group ^157^.

## Final consideration

In vitro ^105^,^158^ and in vivo studies ^5^, ^18^, ^21^ have reported that the current endodontic therapy is unable to eliminate all microorganisms from the root canal system, not only because the influence of anatomical complexities of the pulp space precludes their total extirpation, but also because, inevitably, sufficient nutrients remain to enable most forms of any residual microorganisms to grow. Current technology for mechanical preparation has failed in debriding oval-shaped canals, leaving untouched fins or recesses of the buccal and/or lingual extensions. These untouched recesses may harbor unaffected residual bacterial biofilms and serve as a potential cause of persistent infection and poor treatment outcomes ^105^,^158^. Consequently, these findings emphasize the key role of irrigation and intracanal materials in an attempt to compensate for the suboptimal status of the mechanical debridement throughout the untouched canal areas ^159^. Therefore, efforts have been made to develop novel techniques and devices that provide additional disinfection of the root canal system. The umbrella term for this kind of technique is ‘adjunct therapy’. They involve adjunct treatment steps following traditional cleaning and shaping of the root canal system, aiming at improving root canal cleaning and microbial reduction in order to improve endodontic outcomes ^160^. These adjunct therapies imply a number of different approaches. Some adjunct therapies are based on the use of light, for example, photodynamic therapy ^127^,^128^ and the use of lasers for direct canal wall irradiation or for activation of irrigants ^116^,^117^,^118^. Other techniques use pressure differences to obtain improved cleaning ^141^,^142^. Others make use of vibrating tips in order to improve irrigant streaming, distribution, and action. They include ultrasonic ^18^,^118^ and sonic ^40^ activation. Moreover, other techniques use ozone-generating devices to get the same premises ^68^, ^69^,^70^,^71^,^72^,^73^,^74^. A systematic review on the effectiveness of adjunct therapy for the treatment of apical periodontitis ^114^ aimed to critically appraise all available evidence regarding the efficacy of adjunct therapy in decreasing the occurrence of clinical and radiographic features and contributing to increase the success rate of the endodontic treatment. The authors could not find the superiority of the adjunct therapies evaluated over conventional ones in enhancing root canal disinfection, meaning that up to the moment, the endodontic treatment performed today can reduce the microbial load of the root canals, allowing repair of the periapical lesion. They suggested that more detailed randomized clinical studies are necessary, with a larger sample size in each arm with a long-term follow-up, so that the role of adjunct therapy can be stressed.

Finally, there has been a growing concern to optimize the root canal irrigation step. Regarding irrigants, new alternatives have been developed, but have not yet replaced the existing ones. However, even among the most used irrigants, there is no consensus on the ideal volume and concentration. In addition, countless activation systems were created with the most different action mechanics. However, there is also no consensus on the best system to use for proper cleaning of the root canal system. Furthermore, there is no guideline for the use of time, power, and speed of the different existing drive systems. Therefore, there is a need to standardize the irrigation stage with the most established irrigants and activation systems and, at the same time, a continuous search for the best possible irrigant and activation system. Although it is not possible to conclude that there are clinically proven benefits for adjuvant therapies over conventional methods, it is a promising area in root canal disinfection. Further research is essential in endodontic irrigants and irrigation procedures.

In conclusion,

1) The ideal properties of root canal irrigants have not still been found in one unique substance; 2) The root canal irrigants can be categorized as inactive (inert) irrigants (e.g. distilled water, saline solution) and active irrigants (e.g. NaOCl, CHX, EDTA, natural agents: green tea, Triphala); 3) New irrigant alternatives have been proposed including nanoparticles (silver, chitosan, bioactive glass); ozonated water; 4) Activation system is the process of employing mechanical, physical or another form of energy to agitate and improve the flow of irrigant into the intricacies of the root canal system, in order to improve the removal of smear layer, debris and microorganisms. Conventional Needle Irrigation often fails to deliver and distribute irrigants effectively within a complex root canal system, especially in the apical third and isthmus areas. 5) Among different activation systems instead o activation methods , manual dynamic activation (MDA) is a low-cost technique that does not require additional equipment. It consists of inserting a well-fitted gutta-percha cone, hand files, and brushes, adjusted to the working length in an instrumented canal, using short longitudinal push / pull strokes of 2 mm amplitude at a frequency of 100 strokes in about 1 min; 6) Passive Ultrasonic Irrigation (PUI) is one of the most widely used automated irrigation methods. This method of irrigation is employed following root canal preparation and enlargement. During the ultrasonic activation process, a small diameter non-cutting metal insert is placed in the root canal and must vibrate freely to transmit energy from the tip to the irrigant. It is suggested that for ultrasonically-assisted irrigation to be effective, the tip must operate within a space that is 3 times larger than its diameter. The ultrasonic activation of 1 minute for each canal, with 3 cycles of 20-30 seconds (each with irrigation renewal) has been considered ideal to obtain clean canals; 7) Continuous Ultrasonic Irrigation (CUI) is based on the activation of an insert connected directly to the ultrasonic unit, which allows continuous delivery of the irrigant and simultaneous activation of the insert within the root canal; 8) In the Intermittent Ultrasonic Irrigation (IUI) the irrigant is injected in the root canal with a syringe and it is activated with an oscillating ultrasonic instrument. The canal is filled with the irrigant after each activation cycle. The depth of penetration of the syringe controls the amount of irrigant flushed through the apical region of the canal and the volume of irrigant; 9) Sonic irrigation (SI) differs from ultrasonic irrigation in that it operates at a lower frequency. It consequently produces lower fluid velocity and shear wall stresses, but significantly greater amplitude (horizontal tip displacement). (e.g. Endoactivator, Eddy); 10) Special endodontic files (e.g. Self-Adjusting File, SAF; XP Endo-finisher) are systems that adapt to the walls of the root canal to improve the effectiveness of irrigation and disinfection during chemical-mechanical preparation; 11) Polymer device (e.g. Easy clean), to be used in electric motor-driven instrument in a reciprocal or continuous rotation movement; 12) Light-based Adjunct Therapy is the use of laser light to activate root canal irrigants, called laser-activated irrigation (LAI). In this approach, pulsed laser light is targeting the irrigant within the root canal, to improve irrigant dynamics, distribution, and cleaning action. For this purpose, far-infrared laser light including 2780-nm /2790nm erbium chromium, yttrium scandium gallium garnet (Er;Cr:YSGG laser), or 2940-nm erbium-doped yttrium aluminum garnet (Er:YAG) are typically used. Photon-induced photoacoustic streaming (PIPS) and SWEEPS (Shock Wave Enhanced Emission Photoacoustic Streaming) are examples of LAI Er YAG laser. Antimicrobial photodynamic therapy (aPDT) represents a different light-based adjunct therapy. It is the intracanal application of a photosensitizer (a compound selectively binding microbial cells), followed by irradiation by light whose wavelength matches the absorption peak of the photosensitizer, resulting in a chemical reaction that produces microbicidal elements; 13) Apical Negative Pressure Irrigation (ANPI) is an alternative method for the delivery of irrigants inside the root canal that was proposed in order to minimize the risk of irrigant extrusion through the apical foramen. Irrigants are delivered by a syringe and needle inside the pulp chamber and a fine suction tip placed near the working length creates the necessary negative pressure that drives the irrigant into the canal. Examples of ANPI include Endovac System, CANUI (Continuous Apical Negative-Pressure Ultrasonic Irrigation); iVac™ Apical Negative Pressure Irrigation and Activation System; 14) The RinsEndo device is a mechanism that combines simultaneous irrigation and aspiration under hydrodynamic pressure (positive pressure) 15) The Gentlewave irrigation system is a device developed for cleaning and disinfecting a minimally instrumented root canal. This system uses NaOCl and EDTA, with a rinse of distilled water between them. It applies a Multisonic technology, which means that multiple acoustic frequencies are generated at the same time, and when this technology is paired with optimized procedure fluids, brought about cleaning and disinfection of the entire root canal system, regardless of any anatomical complexities.
